# Clinical characteristics and outcomes of spontaneous bacterial peritonitis caused by *Enterobacter* species versus *Escherichia coli*: a matched case-control study

**DOI:** 10.1186/s12879-016-1595-y

**Published:** 2016-06-07

**Authors:** Seongman Bae, Taeeun Kim, Min-Chul Kim, Yong Pil Chong, Sung-Han Kim, Heungsup Sung, Young-Suk Lim, Sang-Oh Lee, Mi-Na Kim, Yang Soo Kim, Jun Hee Woo, Sang-Ho Choi

**Affiliations:** Department of Infectious Diseases, Asan Medical Center, University of Ulsan College of Medicine, Seoul, Republic of Korea; Department of Gastroenterology, Asan Medical Center, University of Ulsan College of Medicine, Seoul, Republic of Korea; Department of Laboratory Medicine, Asan Medical Center, University of Ulsan College of Medicine, Seoul, Republic of Korea

**Keywords:** *Enterobacter*, Spontaneous bacterial peritonitis, Liver cirrhosis

## Abstract

**Background:**

*Enterobacter* species are important nosocomial pathogens, and there is growing concern about their ability to develop resistance during antimicrobial therapy. However, few data are available on the clinical characteristics and outcomes of *Enterobacter* spontaneous bacterial peritonitis (SBP).

**Methods:**

We retrospectively identified all patients with SBP caused by *Enterobacter* species admitted to a tertiary care hospital between January 1997 and December 2013. Each case was age- and sex-matched with four patients with *Escherichia coli* SBP.

**Results:**

A total of 32 cases with *Enterobacter* SBP and 128 controls with *E. coli* SBP were included. Twenty-one (65.6 %) cases and 111 (86.7 %) controls had Child-Pugh class C (*P* = 0.006). Cases were significantly more likely to have hepatocellular carcinoma (65.6 % vs. 37.5 %, *P* = 0.004) and upper gastrointestinal bleeding (28.1 % vs. 9.4 %, *P* = 0.005). The initial response to empirical therapy (81.3 % vs. 81.2 %, *P* = 0.995) and the 30-day mortality (37.5 % vs. 28.9 %, *P* = 0.35) were not significantly different between the groups. Drug resistance emerged in one case and in no controls (4.3 % [1/23] vs. 0 % [0/98], *P* = 0.19).

**Conclusions:**

Compared with *E. coli* SBP, patients with *Enterobacter* SBP more frequently had hepatocellular carcinoma and upper gastrointestinal bleeding, yet clinical outcomes were comparable. Development of resistance during third-generation cephalosporin therapy was infrequent in patients with *Enterobacter* SBP.

**Electronic supplementary material:**

The online version of this article (doi:10.1186/s12879-016-1595-y) contains supplementary material, which is available to authorized users.

## Background

*Enterobacter* species are important nosocomial pathogens that cause a variety of infections, including bacteremia, pneumonia, surgical site infections, and urinary tract infections [1]. Among Enterobacteriaceae isolated from hospital-acquired infections, *Enterobacter* species have been ranked as the third most frequent isolate following *Escherichia coli* and *Klebsiella* species [2–7]. For more than two decades, there has been growing concern about the induction of β-lactam resistance in *Enterobacter* infections mediated by chromosomally encoded AmpC β-lactamase following antibiotic exposure [8, 9]. This type of inducible resistance is reported to develop at frequencies of 8.3 to 19.6 % during therapy with broad-spectrum cephalosporins for bloodstream infections [10, 11].

*Enterobacter* species are known to be part of the microbial etiology for spontaneous bacterial peritonitis (SBP), being the second to fourth most common causative organism among gram-negative bacilli [12–15]. However, there is a paucity of clinical data about the prevalence, characteristics, and outcomes of *Enterobacter* SBP. Although some small studies included *Enterobacter* species as causative organisms of SBP, the number of included episodes did not exceed five in any study and *Enterobacter* SBP has not yet been analyzed separately. Furthermore, the clinical responses and outcomes of patients treated with third-generation cephalosporins, the most widely used antimicrobials for SBP, have not yet been investigated. Therefore, with a large SBP cohort, we sought to characterize the frequency, clinical manifestations, responses to antimicrobial therapy, emergence of resistance, and outcomes of *Enterobacter* SBP.

## Methods

### Study design, setting, and population

We performed a retrospective case-matched study in Asan Medical Center, a 2700-bed tertiary care institution in Seoul, Republic of Korea. All patients over 17 years who were admitted for SBP between January 1997 and December 2013 were identified by searching for the diagnostic codes “cirrhosis” and “peritonitis” in the computerized database of our institute. Patients with secondary peritonitis, defined as peritonitis caused by perforation or inflammation of intra-abdominal organs, were excluded [16]. Patients who were receiving peritoneal dialysis were also excluded. Finally, we confirmed SBP cases through a detailed review of the medical records. All patients who had *Enterobacter* SBP were included in the study as cases, and they were matched with four-times as many patients with *E. coli* SBP with a similar age (±5 years) and the same sex. If more than four control patients were available for each case, we chose those patients whose dates of admission were closest to that of the case. This study was approved by the institutional review board of the Asan Medical Center.

### Clinical data collection and analysis

We collected the following clinical data for each case and control: age, sex, site of acquisition of the infection, etiology of cirrhosis, comorbid diseases, Child-Pugh score, model for end-stage liver disease (MELD) score, invasive procedures performed within 30 days prior to SBP, antecedent administration of antimicrobials within 30 days, history of past hospital stays before the onset of SBP, initial clinical manifestations, laboratory findings, antimicrobial susceptibility, antibiotic therapy, and treatment outcomes.

In both cases and controls, SBP was subcategorized as either definite or probable. Definite SBP was defined by an ascitic polymorphonuclear cell (PMN) count > 250 cells/mm^3^ and a positive ascitic culture, while probable SBP was defined by an ascitic PMN count > 250 cells/mm^3^ with only a positive blood culture without any other primary focus [17].

### Microbiology

Ascitic fluid samples were inoculated onto blood agar plates, MacConkey agar plates, and Brucella agar plates, and/or the BACTEC system (Becton Dickinson, Heidelberg, Germany). Identification of *Enterobacter* species and antimicrobial susceptibility testing were performed using the Microscan system (Dade Behring, Deerfield, IL, USA). Susceptibility to third-generation cephalosporins was defined according to the revised 2009 guidelines of the Clinical and Laboratory Standards Institute (susceptible, ≤ 8 μg/ml; intermediate, 16–32 μg/ml; and resistant, ≥ 64 μg/ml) [18].

### Statistical analyses

Continuous variables were compared using Student’s *t*-test and the Mann-Whitney *U*-test, while categorical variables were tested by χ^2^ or Fisher’s exact test. To identify clinical factors associated with *Enterobacter* rather than *E. coli* SBP, univariate and multivariate analyses were conducted using a logistic regression model. The clinical variables favoring *Enterobacter* SBP over *E. coli* SBP in the univariate analysis (*P* < 0.1) were included in the multivariate analysis. All *P* values are two-tailed, and *P* < 0.05 was considered statistically significant. SPSS Statistics, version 19.0 (IBM, Armonk, NY, USA), was used for analyses.

## Results

### Demographic characteristics of SBP patients

During the 17 year period of study, a total of 1,172 episodes of culture-proven SBP were identified. *Enterobacter* SBP occurred in 32 patients (2.7 %), including 26 with *E. cloacae* SBP and six with *E. aerogenes* SBP. *Enterobacter* species was ranked as the fourth most frequent pathogen among gram-negative bacilli following *E. coli* (*n* = 536), *K. pneumoniae* (*n* = 196), and *Aeromonas* species (*n* = 62).

The 32 *Enterobacter* SBP patients (cases) were age- and sex-matched with 128 control subjects who had *E. coli* SBP. The demographic characteristics of the patients are summarized in Table 1. In both groups, 81.3 % were male and the mean age was 55 years. The *Enterobacter* SBP patients had a significantly higher percentage of definite SBP cases (81.2 % vs. 60.9 %, *P* = 0.03), hospital-acquired infections (62.5 % vs. 23.4 %, *P* < 0.001), and concomitant hepatocellular carcinoma (HCC) (65.6 % vs. 37.5 %, *P* = 0.004), whereas they had a significantly lower proportion of cases with Child-Pugh score C (65.6 % vs. 86.7 %, *P* = 0.006) and median value of MELD score (19 vs. 23, *P* = 0.03). Antecedent endoscopic interventions had more frequently been performed in the *Enterobacter* SBP group (25.0 % vs. 5.5 %, *P =* 0.001), and the median period between endoscopy and the diagnosis of *Enterobacter* SBP was 22 days (range, 2 − 30 days). Of the eight *Enterobacter* cases involving prior endoscopic intervention, three occurred within 5 days of the endoscopy whereas the remaining five developed more than 3 weeks after the procedure. Anticancer chemotherapy (9.4 % vs. 0 %, *P* = 0.007) and prior exposure to antimicrobial agents (59.4 % vs. 28.1 %, *P* = 0.001) within 30 days were more common in the cases. Multivariate analysis showed that hospital acquisition (OR, 3.19; 95 % CI, 1.25-8.14; *P* = 0.02) and prior endoscopic intervention (OR, 3.57; 95 % CI, 1.02-12.44; *P* = 0.046) were independent factors favoring *Enterobacter* SBP rather than *E. coli* SBP. Results of univariate and multivariate analysis are shown in Additional file 1: Table S1.

### Clinical features and laboratory findings

Table 2 compares the clinical features of SBP and the laboratory findings between the groups. Abdominal pain and fever were the most common initial manifestations in both groups. Upper gastrointestinal bleeding episodes were significantly more frequent in the *Enterobacter* SBP than in the *E. coli* SBP group (28.1 % vs. 9.4 %, *P* = 0.005). Concomitant bacteremia was less common in cases than in controls (34.4 % vs. 68.0 %, *P* = 0.001). The laboratory findings in the *Enterobacter* SBP group vs. the *E. coli* group revealed a trend towards higher levels of systemic inflammatory markers such as serum white blood cell count (median, 8,050 cells/μL vs. 6,450 cells/μL, *P* = 0.12) and serum C-reactive protein (median, 6.41 mg/dL vs. 3.06 mg/dL, *P* = 0.06), but the differences were not statistically significant.

### Antimicrobial susceptibility

Susceptibilities of 31 and 125 clinical isolates of *Enterobacter* species and *E. coli*, respectively, to a range of antimicrobials are shown in Table 3. *Enterobacter* species from 22 patients (71.0 %) and *E. coli* from 106 patients (84.8 %) were susceptible to third-generation cephalosporins and susceptibility of *Enterobacter* SBP group tended to be lower than *E. coli* SBP group (*P* = 0.07). Rates of susceptibility to ciprofloxacin (80.6 % vs. 60.8 %, *P* = 0.038) and trimethoprim-sulfamethoxazole (87.1 % vs. 59.2 %, *P* = 0.003) were higher in the case group, whereas piperacillin/tazobactam susceptibility was lower in the case group (74.2 % vs. 92.8 %, *P* = 0.003). Of the 31 *Enterobacter* SBP patients for whom there were antimicrobial susceptibility test results, 19 (61.3 %) had been exposed to antecedent antimicrobials. Of those 19 patients, 16 had histories of exposure to 3rd-generation cephalosporins. The frequency of resistance to 3^rd^-generation cephalosporins of the *Enterobacter* isolates from patients with prior antimicrobial exposure was twice as high as that in the unexposed group, but the difference was not statistically significant (36.8 % [7/19] vs. 16.7 % [2/12], *P* = 0.42).

### Empirical treatment, outcomes

Antimicrobial treatments and outcomes are summarized in Table 4. The majority of patients in both groups were treated empirically with cefotaxime (84.4 and 90.6 %, respectively). The percentages of appropriateness of the initial therapy were similar between the groups (87.1 % vs. 87.2 %, *P* = 1.00). Treatment outcomes including response to initial therapy (81.3 % vs. 81.2 %, *P* = 0.995) and 30-day mortality (37.5 % vs. 28.9 %, *P* = 0.35) were not significantly different between groups. The lengths of hospital stay were similar between the case group (median, 20 days; IQR, 11 − 31 days) and control group (16 days; IQR, 10 − 26 days, *P* = 0.28). The duration of antimicrobial use was significantly longer in the cases (median, 15 days; IQR, 11 − 25 days) than in the controls (median, 13 days; IQR, 8 − 16 days, *P* = 0.02). There were 2 cases of recurrent SBP in *Enterobacter* SBP group. Among 2 *Enterobacter* isolates from recurrent episode, one was susceptible and the other was resistant to 3^rd^-generation cephalosporins. Antimicrobial susceptibility pattern from those 2 *Enterobacter* isolates were not changed at recurrent episodes.

### Emergence of resistance

A large subgroup of patients was initially treated with third-generation cephalosporins: 27 cases received cefotaxime, and 117 controls received either cefotaxime or ceftazidime. Of these, 23 patients in the *Enterobacter* SBP group and 98 in the *E. coli* SBP group were susceptible to empirical antimicrobial treatment. Emergence of resistance during antimicrobial therapy was identified in one case (4.3 %; 1 of 23) and in no controls (*P* = 0.19).

## Discussion

In this retrospective, case-matched study, we investigated the characteristics and outcomes of the *Enterobacter* SBP. Compared with *E. coli* SBP, patients in the *Enterobacter* SBP group had less severe liver diseases and more commonly had concomitant HCC; they were more likely to have had prior endoscopic interventions and to present with upper gastrointestinal bleeding. However, response to empirical therapy and 30-day mortality rate were not significantly different between the groups. Emergence of resistance to third-generation cephalosporins was infrequent in both groups.

*Enterobacter* species are usually regarded as nosocomial pathogens [1, 19]. According to different studies, 56 − 100 % of *Enterobacter* infections were acquired institutionally [10, 20–23]. Among the 32 *Enterobacter* SBP patients in our study, 20 cases (62.5 %) had hospital-acquired infections; this proportion is within the range of prior reports. Prior antimicrobial usage was also more common in the *Enterobacter* SBP group than in the *E. coli* group. This finding is consistent with previous studies in which prior use of antimicrobial agents was a crucial risk factor for *Enterobacter* infection [1, 10, 24–26]. Interestingly, about two-thirds of the *Enterobacter* SBP group had concomitant HCC; this proportion is significantly higher than in the *E. coli* group, whereas the patients in the *Enterobacter* SBP group had less severe hepatic impairment than the case group. We presume that the treatment of HCC, or HCC *per se*, may contribute to the development of SBP by increasing the probability of hospitalization or antimicrobial exposure.

We found that prior endoscopic interventions were more frequently performed in the *Enterobacter* group than in the *E. coli* group. The rate of transient bacteremia after endoscopic intervention has been reported to be substantial, within the range of 6 − 53 % [27–32]. Bacteremia could lead to subsequent SBP in patients with cirrhotic ascites. A few studies showed that endoscopic interventions for varix control, including endoscopic variceal ligation and endoscopic sclerotherapy, could be complicated by SBP in 0.5 − 3.0 % of cases [32–34]. The interval between the endoscopic intervention and a diagnosis of SBP in those studies was about 2 − 4 days. Of the eight *Enterobacter* SBP patients in our study who received prior endoscopic intervention, three cases were diagnosed with SBP within 5 days and the remaining five cases developed more than 3 weeks after the procedure. Therefore, only the three cases with a short interval between endoscopy and SBP development seem to be directly related to the endoscopic procedure. Intriguingly, we found that upper gastrointestinal bleeding occurred more frequently in the *Enterobacter* group, despite the higher frequency of severe liver disease in the *E. coli* group. Of the nine *Enterobacter* SBP patients with bleeding, seven had HCC, all in an unresectable state. Three of these HCC cases were complicated by portal vein tumor thrombosis and one case by direct duodenal wall invasion of the cancer. Portal vein thrombosis has been considered a risk factor for variceal bleeding [35–37]. We conjecture that more frequent bleeding episodes in the *Enterobacter* group could be explained in part by complications of advanced HCC.

Although susceptibility to third-generation cephalosporins tended to be lower in the *Enterobacter* group, the percentages of appropriateness of antimicrobial therapy, the initial responses to empirical therapy, and the 30-day mortality rates were comparable between groups. The development of resistance during third-generation cephalosporin therapy has been a great concern to clinicians. However, the frequency of resistance arising during third-generation cephalosporin therapy in our study (4.3 %, 1/23) was much lower than those in previous studies regarding *Enterobacter* bloodstream infections (8.3 − 19.6 %) [10, 11]. Cefotaxime, the most widely used initial empiric therapy for SBP in cirrhosis, can penetrate into ascites rapidly and achieve a higher drug concentration in ascites than in blood [38–41]. This pharmacokinetic distinctiveness of the antimicrobial agent could explain the lower rate of emergence of resistance in our study population. Since *Enterobacter* SBP accounted for only 2.7 % of the whole SBP cohort and the treatment outcomes of the *Enterobacter* SBP patients were comparable to those of the *E. coli* SBP, it seems likely that a change of treatment strategy to target *Enterobacter* species is unnecessary.

There are several limitations of this study. First, we conducted a retrospective, case-matched study in a single tertiary center with a limited sample size, which does not permit generalization of our results. Although age and sex were matched in the control group, unexpected confounding factors may have been overlooked. Second, since the isolates were not available, the emergence of resistance during therapy could not be confirmed by molecular methods such as pulsed-field gel electrophoresis. Finally, *E. cloacae* and *E. aerogenes* were analyzed together as a group. The possible differences of antimicrobial resistance pattern and mortality between the two species may have affected antimicrobial susceptibility profile and outcomes in our study [42].

## Conclusions

Our study shows clinical features and treatment outcomes of spontaneous bacterial peritonitis caused by *Enterobacter* species. Compared with *E. coli* SBP, *Enterobacter* SBP was more commonly associated with underlying hepatocellular carcinoma and upper gastrointestinal bleeding. Clinical outcomes associated with SBP were comparable between the groups. Development of resistance during third-generation cephalosporin therapy was infrequently identified.

## Abbreviations

SBP, spontaneous bacterial peritonitis; MELD, model for end-stage liver disease; PMN, polymorphonuclear cell; HCC, hepatocellular carcinoma; IQR, interquartile range; ERCP, endoscopic retrograde cholangiopancreatography; WBC, white blood cells; ICU, intensive care unit.

## Additional file

Additional file 1: Table S1 Univariate and multivariate factors associated with *Enterobacter* spontaneous bacterial peritonitis vs. *E. coli* spontaneous bacterial peritonitis. (DOCX 21 kb)
