# Erratum to: Clinical characteristics and outcomes of spontaneous bacterial peritonitis caused by Enterobacter species versus Escherichia coli: a matched case-control study

**DOI:** 10.1186/s12879-016-1828-0

**Published:** 2016-09-28

**Authors:** Seongman Bae, Taeeun Kim, Min-Chul Kim, Yong Pil Chong, Sung-Han Kim, Heungsup Sung, Young-Suk Lim, Sang-Oh Lee, Mi-Na Kim, Yang Soo Kim, Jun Hee Woo, Sang-Ho Choi

**Affiliations:** 1 Department of Infectious Diseases, Asan Medical Center, University of Ulsan College of Medicine, Seoul, Republic of Korea; 2 Department of Gastroenterology, Asan Medical Center, University of Ulsan College of Medicine, Seoul, Republic of Korea; 3 Department of Laboratory Medicine, Asan Medical Center, University of Ulsan College of Medicine, Seoul, Republic of Korea

## Erratum description

*n.b. The errors and associated corrections described in this document concerning the original manuscript were accountable to the production department handling this manuscript, and thus are no fault of the authors of this paper.*

In the original publication of this article [1], the tables were inadvertently omitted. These can be found below:

**Table 1:** Demographic and baseline clinical characteristics of patients with spontaneous bacterial peritonitis

Variable	*Enterobacter* SBP (*n =* 32)	*E. coli* SBP (*n =* 128)	*P*
Male sex	26 (81.3)	104 (81.3)	
Age, years (mean ± SD)	55 ± 10.9	55 ± 11.7	
SBP subtype			0.03
Definite SBP^a^	26 (81.2)	78 (60.9)	
Probable SBP^b^	6 (18.8)	50 (39.1)	
Place of acquisition			<0.001
Community	12 (37.5)	98 (76.6)	
Hospital	20 (62.5)	30 (23.4)	
Concomitant hepatocellular carcinoma	21 (65.6)	48 (37.5)	0.004
Causes of liver cirrhosis			0.79
Hepatitis B virus	21 (65.6)	88 (68.8)	
Hepatitis C virus	2 (6.2)	13 (10.2)	
Alcoholism	6 (18.8)	19 (14.8)	
Others	3 (9.4)	8 (6.2)	
Child-Pugh class			0.006
A	1 (3.1)	0 (0)	
B	10 (31.3)	17 (13.3)	
C	21 (65.6)	111 (86.7)	
MELD score, median (IQR)	19 (15–24)	23 (18–29)	0.03
Underlying diseases			
Diabetes mellitus	9 (28.1)	25 (19.5)	0.29
Alcoholism	6 (18.8)	23 (18.0)	0.92
Solid cancer (other than hepatoma)	3 (9.4)	4 (3.1)	0.14
Chronic kidney disease	1 (3.1)	2 (1.6)	0.49
Solid organ transplantation	1 (3.1)	0 (0)	0.20
Comorbid conditions			
Transarterial chemoembolization (<30 days)	4 (12.5)	4 (3.1)	0.052
Endoscopic intervention (≤30 days)	8 (25.0)	7 (5.5)	0.001
Varix control	5	6	
ERCP	3	1	
Systemic anticancer chemotherapy (≤30 days)	3 (9.4)	0 (0)	0.007
Prior hospitalization (≤90 days)	26 (81.2)	74 (57.8)	0.01
Prior antimicrobial therapy (<30 days)	19 (59.4)	36 (28.1)	0.001

Values are n (%) unless otherwise indicated. *MELD* model for end-stage liver disease, *IQR* interquartile range

*ERCP* endoscopic retrograde cholangiopancreatography

^a^Ascites culture was positive

^b^Ascites culture was negative, while blood culture was positive without any other primary focus

**Table 2:** Clinical manifestations and laboratory findings of patients with spontaneous bacterial peritonitis by study group

Variable	*Enterobacter* SBP (*n =* 32)	*E. coli* SBP (*n =* 128)	*P*
Initial clinical manifestation, n (%)			
Abdominal pain	23 (71.9)	90 (70.3)	0.86
Fever	24 (75.0)	87 (68.0)	0.44
Hepatic encephalopathy	6 (18.8)	34 (26.6)	0.36
Septic shock	5 (15.6)	31 (24.2)	0.30
Upper gastrointestinal bleeding, n (%)	9 (28.1)	12 (9.4)	0.005
Variceal bleeding	7	11	
Ulcer bleeding	1	1	
Duodenal invasion of cancer	1	0	
Concomitant bacteremia, n (%)	11 (34.4)	87 (68.0)	0.001
Laboratory finding, median (IQR)			
Serum WBC, cells/μL	8,050 (5,125 − 13,200)	6,450 (4,400 − 9,675)	0.12
Platelets, x 10/mL	61 (44–104)	62 (41–85)	0.15
C-reactive protein, mg/dL	6.41 (2.1–7.9)	3.06 (1.1–6.2)	0.06
Serum creatinine, mg/dL	1.2 (0.9–1.5)	1.2 (0.9–1.7)	0.80
Serum bilirubin, mg/dL	3.8 (2.6–12.2)	5.3 (3.5–10.3)	0.79
Ascites WBC, cells/μL	6,160 (1,945–11,795)	5,360 (2,690–11,960)	0.45
Ascites neutrophils, cells/μL	5,058 (1,483–10,899)	4,275 (2,092–10,771)	0.46
Ascites protein, mg/dL	1.5 (1.0–2.5)	1.0 (0.8–1.4)	0.003

*WBC* white blood cells, *IQR* interquartile range

**Table 3:** Antimicrobial susceptibility of isolates of *Enterobacter* species and *Escherichia coli*

Antimicrobial^a^	*Enterobacter* SBP (*n =* 31)	*E. coli* SBP (*n =* 125)	*P*
Cefotaxime	23/31 (74.2)	106/125 (84.8)	0.16
Ceftazidime	22/31 (71.0)	106/125 (84.8)	0.07
Ceftriaxone	22/31 (71.0)	106/125 (84.8)	0.07
Third-generation cephalosporins^b^	22/31 (71.0)	106/125 (84.8)	0.07
Cefepime	25/29 (86.2)	103/123 (83.7)	1.00
Ciprofloxacin	25/31 (80.6)	76/125 (60.8)	0.038
Piperacillin/tazobactam	23/31 (74.2)	116/125 (92.8)	0.003
Imipenem	31/31 (100.0)	125/125 (100.0)	1.00
Trimethoprim-sulfamethoxazole	27/31 (87.1)	74/125 (59.2)	0.003
Amikacin	30/31 (96.8)	124/125 (99.2)	0.36
Gentamicin	27/31 (87.1)	84/125 (67.2)	0.03
Tobramycin	27/31 (87.1)	81/125 (64.8)	0.02

Values are n (%) unless otherwise indicated

^a^Not all of the isolates underwent susceptibility testing

^b^Means cefotaxime or ceftriaxone. The susceptibility to third-generation cephalosporins was defined by the breakpoints of the 2008 Clinical Laboratory Standards Institute guidelines (susceptible, ≤ 8 μg/ml; intermediate, 16–32 μg/ml; and resistant, ≥ 64 μg/ml) [18]

**Table 4:** Treatments and outcomes in patients with spontaneous bacterial peritonitis caused by *Enterobacter* species and *Escherichia coli*

Variable	*Enterobacter* SBP (*n =* 32)	*E. coli* SBP (*n =* 128)	*P*
Initial empirical antimicrobial agent			
Cefotaxime	27 (84.4)	116 (90.6)	0.31
Imipenem or meropenem	4 (12.4)	9 (7.0)	
Cefazolin	1 (3.1)	0 (0)	
Ceftazidime	0 (0)	1 (0.8)	
Cefepime	0 (0)	1 (0.8)	
Levofloxacin	0 (0)	1 (0.8)	
Appropriateness of initial therapy	27 (87.1)	109 (87.2)	1.00
Initial response to empirical treatment^a^	26/32 (81.3)	95/117 (81.2)	0.995
Emergence of resistance during third-generation cephalosporin treatment	1/23 (4.3)	0/98 (0)	0.19
Duration of hospitalization, median days (IQR)			
Overall	20 (11–31)	16 (10–26)	0.28
Survivors	22 (12–31)	16 (11–26)	0.41
Duration of antimicrobial use, median days (IQR)			
Overall	15 (11–25)	13 (8–16)	0.02
Survivors	16 (13–29)	13 (10–16)	0.01
ICU care during admission	2 (6.2)	19 (14.8)	0.25
Mortality			
14-day mortality	7 (21.9)	25 (19.5)	0.77
30-day mortality	12 (37.5)	37 (28.9)	0.35
In-hospital mortality	12 (37.5)	28 (21.9)	0.07

Values are n (%) unless otherwise indicated. *IQR* interquartile range, *ICU* intensive care unit

^a^Ascitic neutrophil decrease >25 % observed 48–72 h after initiating antimicrobials
