# Supplementary material for: Clinical characteristics and outcomes of spontaneous bacterial peritonitis caused by Enterobacter species versus Escherichia coli: a matched case-control study
Source: BMC Infect Dis. 2016 Jun 7;16:252. doi: 10.1186/s12879-016-1595-y (PMC4897898; doi:10.1186/s12879-016-1595-y)
Supplement: Additional file 1: Table S1 — Univariate and multivariate factors associated with Enterobacter spontaneous bacterial peritonitis vs. E. coli spontaneous bacterial peritonitis. (DOCX 21 kb) [file 12879_2016_1595_MOESM1_ESM.docx]

**Table S1. Univariate and multivariate factors associated with *Enterobacter* spontaneous bacterial peritonitis vs. *E. coli* spontaneous bacterial peritonitis.**

| Variable | Results of  univariate analysis | |  | Results of  multivariate analysis | |
| --- | --- | --- | --- | --- | --- |
|  | OR (95% CI) | *P* |  | OR (95% CI) | *P* |
| Hospital acquisition | 5.44 (2.39-12.42) | 0.000 |  | 3.19 (1.25-8.14) | 0.02 |
| Concomitant HCC | 3.18 (1.41-7.17) | 0.005 |  | 2.23 (0.86-5.79) | 0.10 |
| Endoscopic intervention (≤ 30 days) | 5.76 (1.90-17.39) | 0.002 |  | 3.57 (1.02-12.44) | 0.046 |
| Prior antimicrobial Tx. (< 30 days) | 3.74 (1.67-8.34) | 0.001 |  | 2.60 (0.99-6.83) | 0.053 |
| TACE (< 30 days) | 4.43 (1.04-18.79) | 0.044 |  | 3.13 (0.60-16.42) | 0.18 |
| Prior hospitalization (≤ 90 days) | 3.16 (1.21-8.21) | 0.02 |  | 1.15 (0.39-3.49) | 0.79 |

HCC, hepatocellular carcinoma; TACE, transarterial chemoembolization.
